# Controlled Sensing of User-Defined Aptamer-Based Targets Using Scanning Ionic Conductance Spectroscopy

**DOI:** 10.1021/acsnano.4c18509

**Published:** 2025-03-31

**Authors:** Helena Miljkovic, Lely Feletti, Gordanna Pistoletti Blanchet, Marcos Penedo, Zahra Ayar, Barney Drake, Alexandre Kuhn, Wayne Yang, Georg E. Fantner, Aleksandra Radenovic

**Affiliations:** † Laboratory of Nanoscale Biology (LBEN), Institute of Bioengineering, School of Engineering, Swiss Federal Institute of Technology Lausanne (EPFL), 1015 Lausanne, Switzerland; ‡ NCCR Bio-Inspired Materials, École Polytechnique Fédérale de Lausanne, 1015 Lausanne, Switzerland; § Laboratory of Molecular Biology, Institute of Life Sciences, School of Engineering, HES-SO Valais-Wallis, 1950 Sion, Switzerland; ∥ Laboratory for Bio and Nano Instrumentation (LBNI), Institute of Bioengineering, School of Engineering, Swiss Federal Institute of Technology Lausanne (EPFL), 1015 Lausanne, Switzerland

**Keywords:** aptamer, protein, single molecule detection, solid-state nanopore, SICS

## Abstract

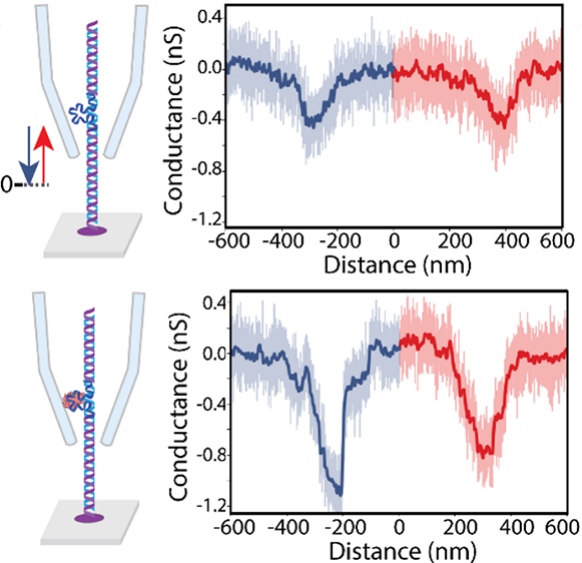

Solid-state nanopores offer the possibility of detecting disease biomarkers in early diagnostic applications. Standard approaches harness fingerprinting, where protein targets are bound to DNA carriers and detected in free translocation with a solid-state nanopore. However, they suffer from several drawbacks, including uncontrolled fast translocations, which lead to low detection accuracy and a low signal-to-noise ratio (SNR). This has hampered their application in clinical settings. Here, we propose a nanopore-based system capable of sensing selected molecules of interest from biological fluids by harnessing programmable aptamer sequences attached to DNA carrier systems that are tethered to glass surfaces. This allows for spatial and velocity control over translocation in the *x*, *y*, and *z* directions and enables the repeated scanning of the same analyte. The scanning ion conductance spectroscopy (SICS) based approach distinguishes itself from standard nanopore-based approaches with its ability to repeatedly scan the same aptamer molecule target site more than 5 times. We designed a DNA carrier with multiple binding sites for different aptamers to increase the yield of the experiment. Our approach achieves a detection rate of up to 74%, significantly higher than the 14% achieved with standard solid-state nanopore measurements. The strong spatial control also allows for significantly increased densities of aptamer target sites along the same DNA carrier, thereby paving the way for multiplexed sensing. The system offers user-defined programmability with different aptamer sequences, potentially expanding the use of our system to sense other disease biomarkers.

## Introduction

Single-molecule sensors have demonstrated the ability to identify and quantify proteins for various diagnostics applications.^1−4^ In such cases, the analyte of interest passes through a tiny aperture (also known as a nanopore) and the resulting characteristics of the ionic blockade signal of the molecule provide information on its volume, charge, and conformation in a label-free manner.^5,6^ Using this principle, nanopore sensing has been employed to successfully detect a wide range of biological constructs, including dsDNA,^7−9^ ssDNA,^10,11^ RNA,^12−15^ proteins,^4,16−24^ and DNA-binding proteins^25−28^ on both solid state^5^ and biological^29,30^ platforms.

Significant challenges remain in nanopore sensing using solid-state nanopores–in particular, the translocation events display low signal-to-noise ratio (SNR).^2,31^ This makes analysis challenging as the signature signal of the protein often becomes averaged out or obscured by electronic noise.^22^ Additionally, nanopore sensing largely relies on stochastic diffusion processes to capture the analyte, particularly in the case of neutrally charged proteins, leading to low capture rates.^32^ Alternately, one can harness electroosmotic flow (EOF), which drives molecules through the flow of counterions.^33,34^ However, such approaches require the use of concentration gradients,^32^ mesh networks,^35,36^ low pH buffers,^33^ highly negative DNA origami structures,^37^ or modified protein pores^34^ which complicates the experiments. The problem is compounded when scaling to human-derived samples which contain a large variety of proteins present in the solution, leading to issues with clogging and false-positive identification of the biomarker of interest.^5,38^ In recent years, some approaches have relied on “DNA carriers” to precapture the analyte of interest within the solution and capture it for interrogation.^4,39^ These carriers consist of long dsDNA hybridized with various constructs, including RNA,^12,39,40^ DNA origami strands,^41^ CRISPR/dCas9,^28,27,42^ and aptamers allowing specific targeting.^4,39^ The presence of the targets is usually marked by the presence of current decreases within the ionic current trace resulting from the excluded volume of the target binding. However, these methods are still limited by the free translocation of the DNA carriers, with large variability in their current signature. Variability arises from uneven translocation velocities^43,44^ and different DNA topologies^45^ (such as folds, which manifest as increased blockade current), leading to low detection rates (∼10–30%) or false positive events.^3^ Typical solutions to this problem include only selecting “linear” translocation events, whereby the DNA is captured without any folds.^45^ Other efforts to control such translocation include coupling the same DNA strand between two or more nanopores to place the molecule under tension, thereby linearizing it and flossing the same molecule back and forth to resense it.^46,47^ However, large capacitances and coupling of the two electric fields of the nanopores obscure the readout and detection.

**Figure 1:**
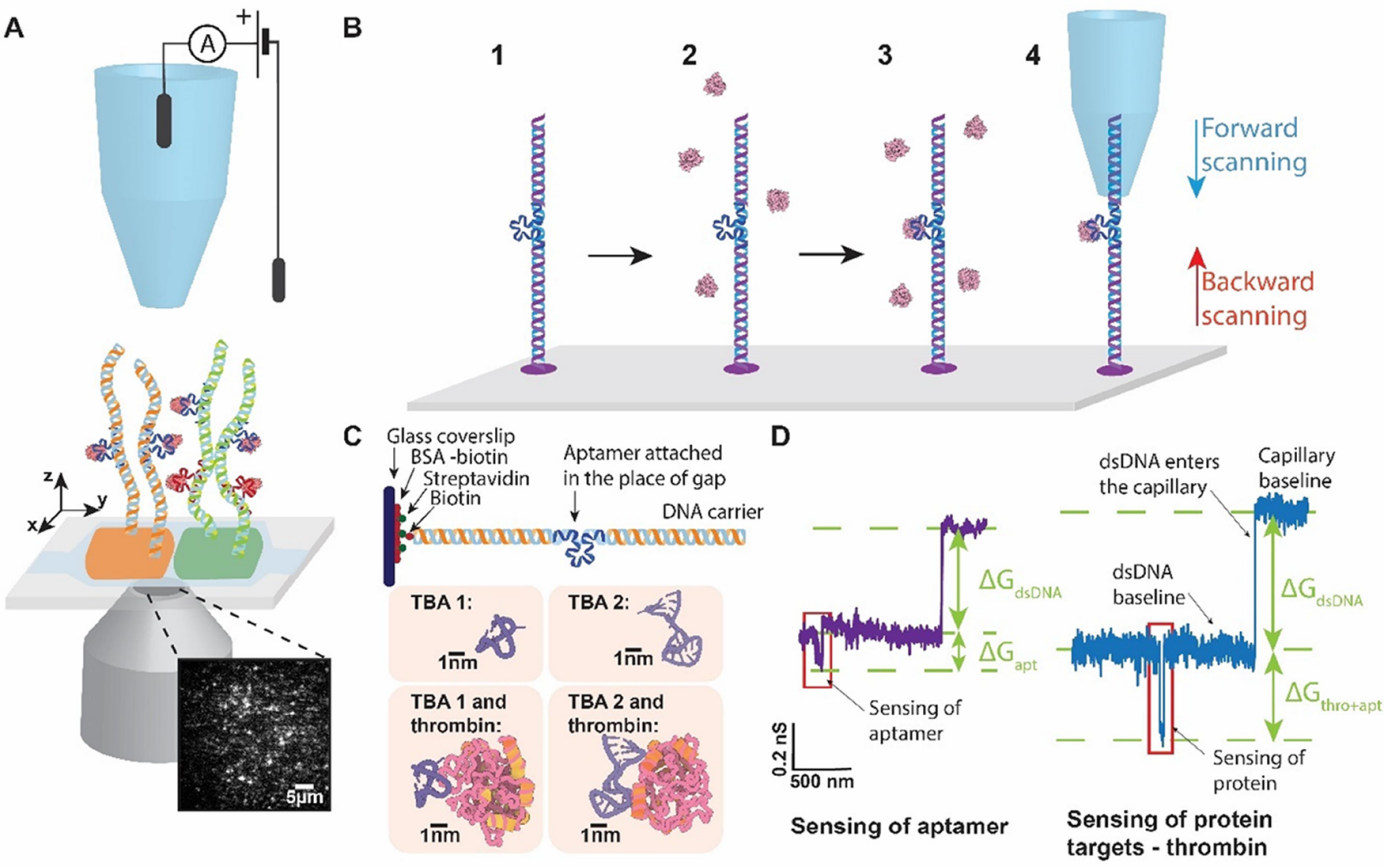
Scanning ionic conductance spectroscopy of DNA carriers with aptamers. (A) Illustration of the SICS system. The system consists of multiple chambers containing different programmable DNA carriers. A bright field and fluorescence microscope are aligned, allowing us to track the capillary’s position as well as the location of molecules tethered to the surface. We show a sample fluorescence image with labeled DNA (YO-PRO-1 dye), showing the location and density of the immobilized molecules. (B) Illustration of the sensing protocol. In **step 1** - DNA carriers with aptamer are incubated onto the surface. In **step 2** - protein is introduced into the buffer, where it is subsequently attached to the aptamer sites (**step 3**). In **step 4** - the SICS is approached and the DNA carrier is scanned linearly to detect the presence of the target protein. (C) Design of DNA carrier with 1 gap and its attachment to the surface and the corresponding crystal structure and shape of the target analyte used in this work. An illustration of the structures of aptamers TBA 1^57^ and TBA 2,^61^ along with their specific binding interactions with thrombin.^62^ (D) Example current traces of aptamer and of thrombin on 1 M KCl and 300 mV voltage. The thrombin target shows up as a much larger current blockade owing to its increased size when compared to the aptamer only site.

Here, we present a strategy that attaches one end of the DNA carriers with user-defined aptamer targets to a surface for interrogation with scanning ion conductance spectroscopy (SICS) (i.e., a movable nanopore, Figure 1A). SICS is a nanopore-based sensing method that enables the translocation of analytes at a controlled speed.^42^ This control is achieved by introducing a movable solid-state glass capillary of adjustable size and fixing the analyte onto the surface. We previously demonstrated that this approach circumvents the reliance on stochastic capture and allows for controlled spatial interrogation of DNA gaps. Building on this technique, we now employ DNA carriers with ssDNA gap sites that allow for aptamer binding, leading to new avenues for multiplexing and repeated rescanning of bound analytes with user-defined velocity for improved detection. We show more than a five-fold increase in detection rates (74%), which is limited by analyte binding. Moreover, we demonstrate specific advantages of SICS, such as controlled translocation speed, 3D spatial multiplexing, high SNR, and rereading capabilities for improved detection. The system’s specificity and sensitivity show significant potential for diagnostic and therapeutic applications.

## Results

The SICS-based detection scheme makes use of long DNA carriers modified to accommodate aptamers for targeted proteins, both with and without the bound protein (Figure 1B). After introducing proteins that bind to the DNA carrier construct, a nanopipette scans along the direction of the DNA carrier for positive identification of the protein's presence. The DNA carrier consists of a long 8750 bp dsDNA with a 40-base long single-strand part (gap) in the middle, enabling user-defined aptamer sequences to be bound (see Figure S1A). As a proof of principle, we employ two antithrombin aptamers: the 15-mer TBA 1^48,49^ (5′-GGTTGGTGTGGTTGG-3′) and the 29-mer TBA 2^50^ (5′-AGTCCGTGGTAGGGCAGGTTGGGGTGACT-3′). These two aptamers were selected for the wide range of dissociation constants (i.e., for TBA 1 is 10–100 nM,^51^ and for TBA2 is 100–500 nM^50,51^) to demonstrate the versatility and working range of our strategy. The stability of the aptamer was verified using a gel shift assay (see Figure S1B,C). To bind the DNA carriers, we employed biotinylated ends of the DNA (Figures 1C and S1A) to immobilize the entire DNA-aptamer construct on a streptavidin functionalized glass surface. We checked the immobilization of the DNA carrier on the surface and imaged it with fluorescence microscopy (Figure 1A insertion). In the follow-up experiments, we modified the same DNA carriers to include an additional gap, allowing for the binding of multiple aptamers to the different gap sites.

### SICS Experiments

**Figure 2:**
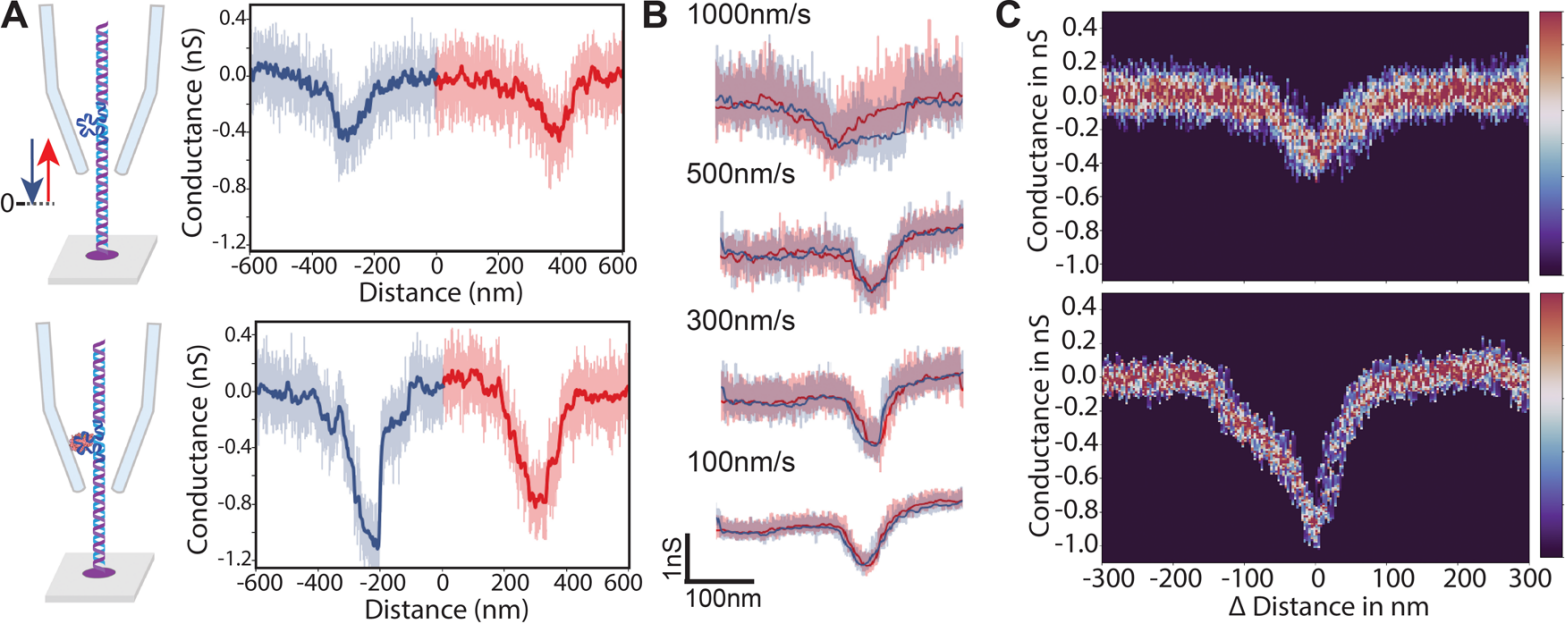
Example current traces showing forward scanning and rescanning of DNA carriers. (A) Example current trace showing forward (blue) and backward (red) scanning of the aptamer (top) and thrombin (bottom). (B) Current traces as a function of pipet speed. We notice a trade-off between faster scanning speed and lower signal-to-noise ratios. In the rest of the study, we employed a scanning speed of 300 nm/s. (C) Overlaid density plot of sample current traces. We overlaid a density plot of aptamers (top, *N* = 6) and bound thrombin (bottom, *N* = 6). The traces showed remarkable consistency and overlap.

We performed initial SICS experiments to demonstrate the sensing of DNA carriers prior to building statistics. Briefly, we followed the same procedure as fully described in Leitao et al.^42,52^ Quartz glass capillaries with nanopore diameters of 25–40 nm (Figure S2) are mounted on a step motor, while the sample is placed onto the piezo stage. By synchronizing the movements of these two devices, the capillary can be controllably approached to the surface with high precision. The size of the capillaries was selected to match the analyte, being slightly larger to optimize the SNR. The capillary was filled with the sensing buffer (1 M KCl) and brought into contact with a sample buffer. A voltage is applied across the aperture of the glass capillary, and the open pore current of the capillary is recorded. When the capillary approaches the surface, part of the ionic current is blocked, leading to a decrease in current. This change indicates the position of the surface with respect to the entrance of the capillary and allows precise advancement toward the sample. When the nanopore captures a DNA carrier, we observe a decrease in the current, establishing a new DNA baseline current (which we note as ds-DNA current, typically in the range 0.4–0.7 nS, in 1 M KCl at 300 mV, as shown in Figure 1D). As the capillary moves further along the DNA carrier, it reaches the location of the DNA gaps where the aptamers are bound (in a forward direction, see Figure 2A). There, we observe a further blockade owing to the larger excluded volume (typically an additional 0.4 nS for our aptamers). This process can also be repeated as the capillary is retracted in the backward direction (see Figure 2A). Taken together, these two pieces of evidence confirm the sensing of conjugated aptamer constructs.

One of the advantages of the setup is its ability to directly control the translocation speed (unlike that in conventional nanopore sensing). We performed the translocation of DNA carriers with aptamers at different translocation speeds to boost the SNR. Figure 2B shows the signal obtained at different translocation speeds of 100, 300, 500, and 1000 nm/s. We see an increase in SNR at slower scanning velocities (e.g., 100 nm/s) at the cost of lower rastering rates across the entire sample. After comparing the SNRs, we selected a translocation speed of 300 nm/s for all subsequent scans, as it offered a good compromise between SNR and acceptable rasterizing rates across the sample, thereby improving statistics.

Subsequently, we tested DNA-aptamer constructs for their ability to capture thrombin with a glass capillary. As described above, a similar procedure was performed for sensing along the DNA carrier. Now, in place of the aptamer signal, we notice a larger conductance change (typically 0.9–1.6 nS vs 0.3–0.6 nS from the aptamer-only condition) from the thrombin attachment (see Figure 2C for overlaid density plot).

### Statistics of DNA Carriers Containing a Single Aptamer

**Figure 3:**
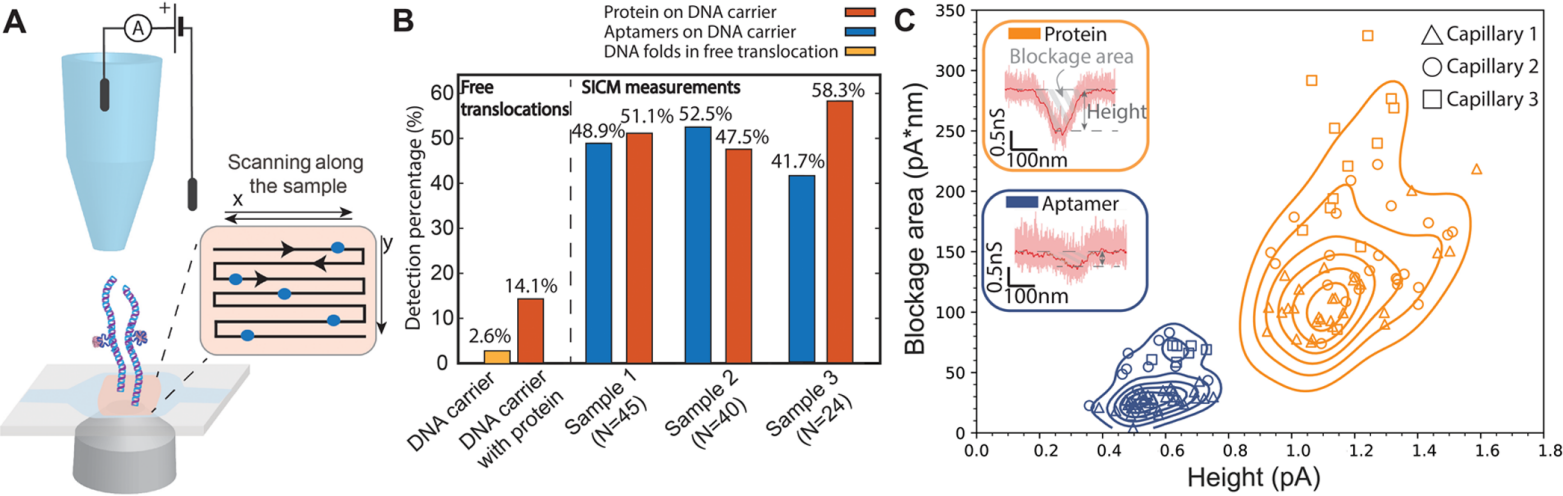
Verification and repeated measurement on sample. (A) Illustration of the scanning pathway taken by the SICS. The sample is scanned for multiple molecules by rastering the pipette across the sample in a snake-like manner (first along an *X* direction (continuously scan) and then a shift in *Y*, stepwise 1 μm). (B) Histogram of data collected for three different samples compared with control experiments in free translocation. We achieve significantly higher rates of detection with our SICS based approach when compared to free translocation experiments. (C) Contour plots of the integrated current blockade vs height of the signal from 3 different capillary measurements (*N*_cap1_ = 23, *N*_cap2_ = 72, *N*_cap3_ = 39). We observe 2 different clusterings of the signal which we associate to either the presence of the aptamer only or thrombin bound conditions. Insertion: Each sensed molecule was characterized by measuring its height and blockage area.

SICS detection of thrombin yields a higher detection rate compared to conventional nanopore sensing. Each independent DNA carrier was repeatedly scanned (2–3 times along the gap position) before the capillary was fully retracted and moved to scan a new DNA carrier (Figure 3A). This allowed us to build statistics on the binding rate in our sample (see Figure S3). The signal was sorted into two categories as marked by their blockade level (described previously): (i) aptamer and (ii) thrombin bound. Here, we did not detect any DNA carriers without a bound aptamer. We repeated our measurements on three different samples to ensure reliability. Figure 3B shows the binding rate of both categories across three independent samples (*N* = 45, *N* = 40, *N* = 24). The sample was characterized based on the height and area of the current blockage caused by the molecule detected along the DNA carrier (Figure 3C, inset). A clear distinction was observed between the aptamers alone and the aptamers bound to the thrombin protein even with different sizes of capillaries, indicating successful protein attachment (see Figure 3C). We detected thrombin bound to our DNA carriers (52 ± 5)% of the time. As a control, we performed free translocation measurements with the similar size quartz nano capillaries (1 M KCl, 300 mV) of the DNA carriers with aptamers and thrombin incubated identically. The free-translocation experiments yielded only 14.1% events with a deep blockade which we identified as thrombin-bound (see Figure S4(53)). Overall, SICS detection demonstrates a 3X improvement in the detection sensitivity of the target molecules compared to free translocations.

### Multiplexing in the *z* Direction

**Figure 4:**
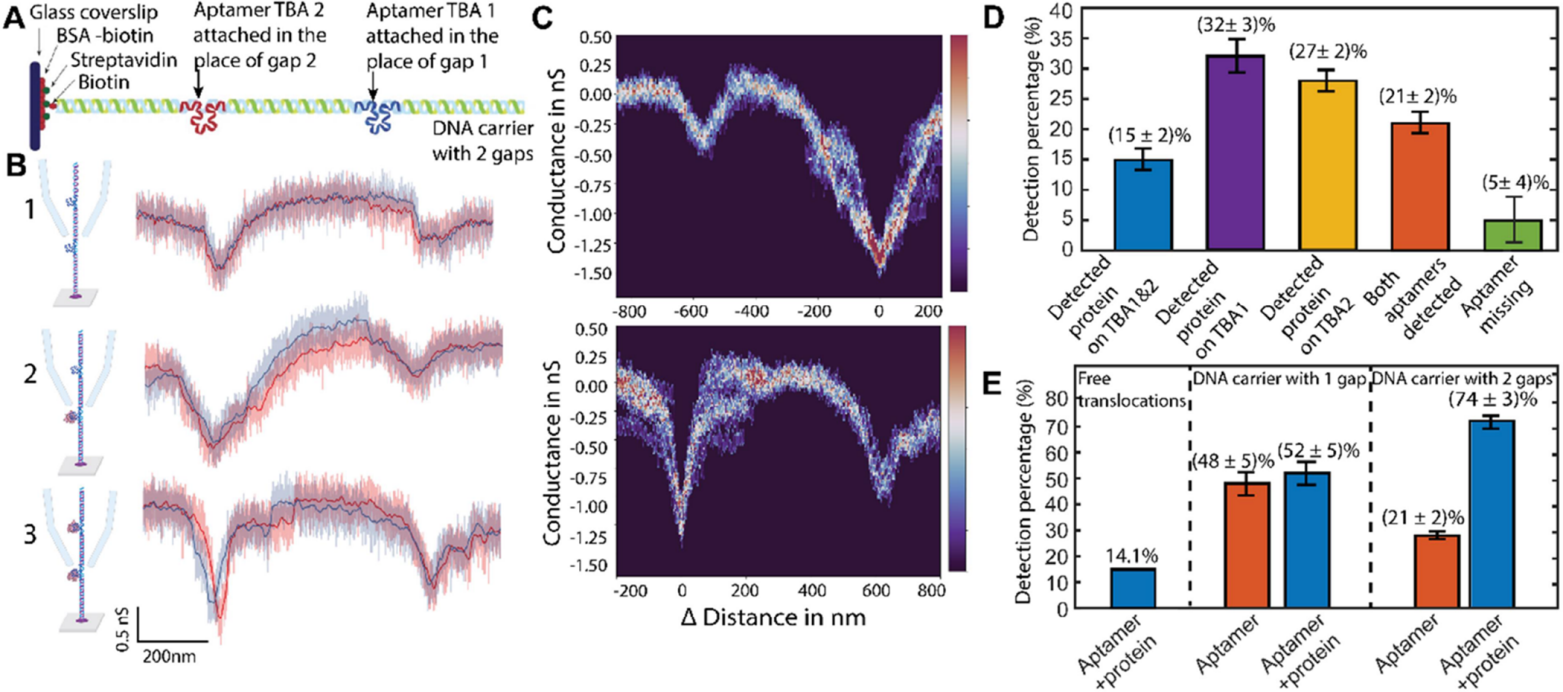
Multiplex DNA carrier experiments. (A) Design of a DNA carrier with 2 gaps and attachment to the surface. (B) Examples of measurements done on DNA with 2 gaps and sample current traces showing 3 different cases. **Case 1**: both gaps containing aptamers, in gap 1 is TBA1, in gap 2 is TBA2 **Case 2**: both gaps containing aptamers but only one site (TBA2) contains an attached protein. **Case 3**: both gaps containing aptamers and both sites with attached proteins. (C) Density function plot of repeated measurements with the same capillary (*N* = 6). Top: Density function plot of the molecules that are thrombin bound only in gap 1 (TBA1). Bottom: Density function plot of the molecules that have thrombin-bound in both gaps 1 and 2. (D) Histogram of repeated measurements showing percentages of events sorted by the 5 different cases (*N* = 3). (E) Aggregated data from repeated experiments. Overall binding percentages as detected by the SICS approach is shown in the 2 different cases (aptamer and aptamer bound to the thrombin). We achieve a much higher detection rate with the 2 gap system.

SICS allows for the sensing of multiple analytes along the same DNA carrier (Figure 4) with unprecedented spatial resolution. To take advantage of this feature, we modified a DNA carrier to include a second gap facilitating the binding of another aptamer (Figure 4A). We selected another thrombin-binding aptamer–TBA 2, with a dissociation constant of a different magnitude than TBA 1 (i.e., for TBA 1 is 10–100 nM,^51^ and for TBA 2 is 100–500 nM^50,51^) as a proof of concept. We used the same DNA carrier construct as described above but with an additional 40 bp gap located at a distance of 1030 bp from the first. The DNA carrier sample was checked without any aptamers in order to localize the gaps. The translocation signal of the DNA carrier-only sample yielded a distinct two-bump feature (a double hump in the ionic conductance signal of typically 0.7 nS). These double peaks were observed to be 450–600 nm apart, as measured by the SICS piezo scanner. This roughly corresponds to the expected genomic distance between the gaps in our construct (∼370 nm for 1030 bp with a base pair spacing of 0.34 nm). The increase in the length of DNA has been observed in a variety of other nanopore sensing studies and it is attributed to the applied tension on the DNA in combination with the effective screening of the DNA bonds in the high-concentration salt buffer.^13,54,55^ This also provided a rough indication of where along the *Z* axis we will localize TBA 1 and TBA 2. TBA2 was attached to the gap closer to the biotin-end (i.e., the end closer to the surface), while the one further along from the end was attached with TBA 1. We hybridized both aptamers simultaneously in the same incubation step, following the same protocol as the DNA carrier with one gap (see the Method section for the full sequences including the complementary DNA sequences for binding). We speculate that it will be possible to similarly hybridize multiple different aptamers simultaneously, in the same one-step reaction, for future multiplexing in the *z* direction.

### Statistics of DNA Carriers Containing Two Aptamers

SICS detection of thrombin on DNA carriers with two gaps (Figure 4A) further improves the detection rate of the proteins. After detecting the entry of DNA into the capillary, we scanned the capillary along the DNA to the positions of the two gaps. At both expected gap positions, we observed distinct decreases in current–similar to the single DNA gap case, indicating thrombin binding (Figure 4B). We used the same criteria as in the single-gap case, detecting aptamer for a smaller additional dip (typically additional 0.4–0.7 nS) and thrombin for a larger dip (typically 0.9–1.6 nS) (see Figures 4C and S5). We independently scanned multiple molecules along the surface in order to build statistics on the binding rate for the sample. The detected signal at the gaps was sorted into 5 categories based on their blockade level: (i) thrombin on both TBA 1 and TBA 2 (ii) thrombin only on TBA 1 (iii) thrombin only on TBA 2 (iv) both aptamers bound (v) one or both aptamers missing with no proteins bound. Figure 4D shows the percentage distribution for each respective category. We observed a difference in binding rates between the TBA 1 and TBA 2 aptamers (32% vs 27%). Given that our measurements were conducted in a buffer with high protein concentration, these differences are attributed to factors such as folding and the stability of the aptamers in the high-salt buffer.^56,57^ When the binding rates of the two gaps are combined, we achieve a detection percentage of 74% for detecting thrombin binding across 3 different repetitions (*N* = 43, *N* = 31, *N* = 19). Comparing the detection rates of single-gap DNA carriers and two-gap DNA carriers, we observed an improvement from 52 to 74% (Figure 4E). Here, we suspect that the binding rate of the aptamer is a limiting factor for the improvement of these measurements.

### Measurements in Environmental Conditions and Human Serum

**Figure 5:**
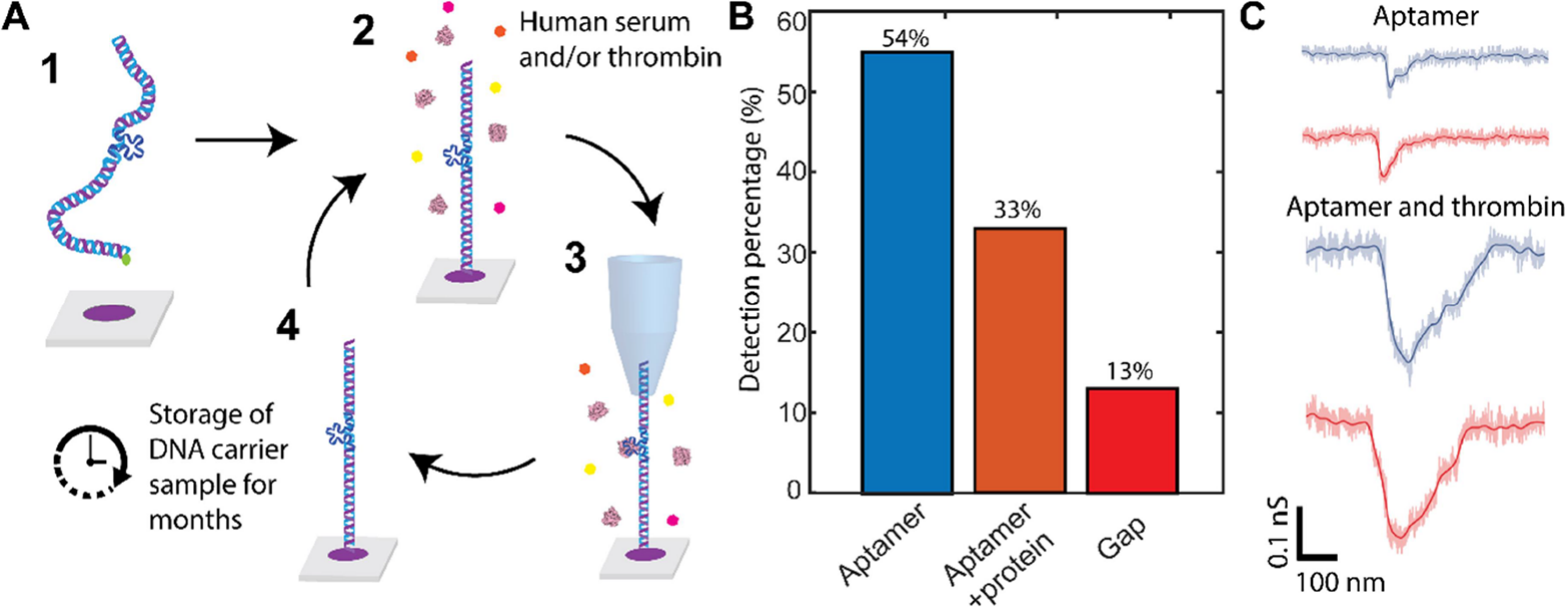
Measurements in environmental conditions and human serum. (A) Sensing protocol for detecting thrombin in human serum as mimics for a clinical sample. **Step 1**: incubation of the DNA carriers onto the surface. **Step 2**: introduction of target protein in human serum. **Step 3**: sensing of the thrombin molecule. **Step 4**: rinsing of the sample. After this step, we can introduce the new buffer again for repeated measurement. (B) Histogram of repeated measurements showing percentages of events showing attached or not attached protein to the DNA carrier (*N* = 30). (C) Example measurements of aptamer and thrombin bound to aptamer 150 mM KCl buffer. The sample has been used after 5 months and still has aptamers attached.

Motivated by these efforts, we explored our system for sensing with samples mimicking clinical samples and tested the robustness of the SICS approach. We envision the following process: clinical samples containing the disease biomarker are flushed through our preincubated DNA-aptamer carriers. To mimic such a process, we employed human serum as the starting buffer. Human serum introduces new challenges because it contains a wide variety of proteins (such as immunoglobulins, albumin, hormones, etc.) that may overwhelm the signal. Thrombin, representing our target analyte, was dissolved in human serum in order to form a mimic (thrombin was incubated in twice-diluted human serum and 1 M KCl). The SICS detection platform was prepared by incubating the immobilized DNA carriers with the antithrombin aptamer TBA 1 (i.e., the aptamer with the higher measured detection rate). We utilized only the single-gap capture strand in our proof-of-concept experiment (Figure 5A **step 1**). Next, we replicated a real-world detection process by flowing the human serum containing the thrombin target through our DNA-aptamer sample (Figure 5A **step 2**). The sample was left to incubate for 30 min, after which it was probed (Figure 5A **step 3**). The sample was reused for measurements across eight different days (Figure 5A **step 4**). After each session, it was rinsed and stored in a refrigerator at −4 °C. This step allows us to introduce the new buffer and reincubate the sample for the next experiment session. As expected, the high affinity of thrombin to the aptamer led to clear detection by SICS. Using the same detection criteria, we detected >30% of molecules with attached thrombin (Figure 5B). We also extended our studies to sensing with 150 mM KCL, a much lower concentration closer to clinical conditions thereby expanding the library of aptamers that can be used (Figure 5C).^58^

## Discussion

Overall, our results show that SICS circumvents some current limitations in nanopore measurements for the detection of proteins under physiological conditions. By taking advantage of a fixed analyte and making the nanopore movable, we are able to reliably sense DNA carriers rather than rely on the stochastic capture of the analyte. Our system offers several advantages over standard nanopore approaches. First, we achieve capture and resensing of the same molecule, allowing for a much higher detection rate. Second, we spatially separate and define different multiple sensing sites along the same DNA carrier, which opens avenues for multiplexing. Using a multiplexing approach where two binding sites are present along a DNA carrier–we demonstrate a high percentage of detection of over 74%, which strongly suggests that the detection limiting step now lies in the binding affinity of the aptamers rather than the nanopore detection approach. Third, we demonstrate the long-term stability of our approach and the repeated use of the same carriers for multiple samples over extended periods. Fourth, we successfully sensed aptamers and proteins in human buffers and physiological conditions (150 mM KCl). The approach reported here marks a significant improvement over previous nanopore approaches in multiple ways. Complications such as DNA topology and folding, which are typical in conventional nanopore translocation experiments, are no longer observed. Previous works dealt with this problem by filtering and selecting only linear events (type 1 events),^45^ representing only 30% of the data collected. This leads to low positive detection rates due to low SNR or analyte unbinding in high-salt buffers. Additionally, by controlling the speed of the scanning nanopore, we minimized the interaction of thrombin with the capillary (a common issue faced by normal nanopore measurement), allowing for more accurate determination of the spatial positioning and amplitude of the current blockade produced by the target analyte. This boosts the SNR and increases the sensitivity of our SICS approach, thereby allowing us to operate under physiological-like conditions. Our approach also provides a key advantage for multiplexing since the aptamer sites along the DNA carrier can be spatially distinguished by SICS. The aptamers employed in this work were spaced ∼1000 bp apart, allowing clear separation and identification of the 2 sites in the ionic current signal. In principle, it is possible to cover the entire length of the DNA carrier with the same density, leading to 5 sites along the same DNA strand. Higher densities can also be achieved by extending the carriers past the typical persistence length of the carriers as our capillary linearizes the DNA upon capture prior to scanning. The drawback of this method is that it can detect only one molecule at a time, which increases the detection time compared to other methods, especially when probing a large sample size. We hypothesize that presorting and upconcentrating the sample with nanofluidics or microfluidics systems in the upstream process will ensure a more representative sampling of clinical samples without sacrificing sensitivity benefits with our proposed SICS method.

Looking ahead, our approach could potentially allow us to observe dynamics on the nanoscale, which might be exploited in future sensing applications. Aptamer structures are rigid, which results in very defined and sharp ionic current peaks.^59^ The bound proteins, on the other hand, seem to fluctuate around their bound sites and position during the scanning of the capillary. We believe that both conformational change of the protein and interactions with the capillary cause such fluctuations. As already observed in our experiments, repeated measurements of the same molecule yield different possible orientations and positions of the thrombin protein in the capillary. We speculate that it will be possible to probe other protein–DNA complexes in the same way to measure their resulting impacts on their current traces and reveal their influence on the surrounding ionic environment.

## Conclusions

In summary, using SICS, we developed a strategy for protein detection with controlled translocations of DNA carriers featuring user-defined aptamer targets attached to a surface. Our detection probe is a solid-state glass capillary, which enables a controlled approach, capture, and scanning of individual DNA carriers attached to the surface. This method eliminates reliance on the stochastic movement of analytes in buffer solutions, allowing for the controlled spatial interrogation of the carriers, and opens new possibilities for multiplexing. We demonstrate a proof of concept with thrombin-binding aptamers TBA 1 and TBA 2. We achieve high detection sensitivity with our SICS approach, surpassing previously reported values in the literature. Additionally, by concatenating and increasing the number of binding sites, we showed a more than five-fold increase in detection rates (74%), with the primary limitation being the analyte binding efficiency. By applying this system to the detection of thrombin in human serum, we demonstrated the potential of the platform to be used as a method of detection in a system with a protein mixture. It will be possible to use our approach and other relevant aptamers to find different biomarkers for disease detection. We believe that this technology further extends nanopore-based approaches for use as diagnostic tools and in single-molecule studies.

## Method

### Experimental Setup

#### SICS System and Controlled Translocation Measurements

SICS is a nanopore-based measurement system for controlled sensing of *XY*-spatially multiplexed molecules. To approach the surface, we used synchronized motion in the *z*-direction via a step motor and a piezo stage. During the approach, a trigger is activated to stop movement if the measured ionic current decreases to 99.5% of its initial value. In case a threshold is not reached through the full *z*-height range of the piezo stage, it retracts and a step is made by the step motor. This setup allowed us to progressively assess the proximity to the surface using the piezo stage, followed by stepwise advancement with the step motor. Once the threshold is reached, the capillary is in the approached position (in reference to the surface) and stops moving. Once we approached the surface, a positive voltage was applied to the capillary and the distance from the surface was modulated for effective capture of the DNA. These parameters are highly dependent on the salt concentration of the buffer. When the molecule was captured, it could be sensed at a controlled speed and resensed multiple times. By moving capillaries in the *XY*-direction, we were able to analyze different molecules and build statistics on them.^52,42^ All displayed current traces include a light-shaded line, which is the downsampled signal at 1 kHz, and a bold line, representing the Gaussian-filtered signal.

### Probe Preparation

#### Nanopipette Fabrication and Filling Protocol

Quartz capillaries (Hilgenberg GmbH, length 70 mm, outside diameter 0.5 mm, and inside diameter 0.2 mm) were pulled by using a CO2 laser puller (P-2000, Sutter Instruments) with the following protocol: Heat = 580, Fil = 0, Vel = 15, Del = 170, Pul = 0; Heat = 570, Fil = 0, Vel = 15, Del= 170, Pul = 200. Nanopipettes were then placed inside a plasma cleaner and immediately immersed in the chosen buffer solution. They were subsequently left for 15 min in a desiccator and microwave heated for 3–6 min, which eliminated the bubbles from the nanopipettes and completely filled them with buffer solution. Nanopipettes fabricated in this way have a diameter ranging from 25 to 40 nm.

### Sample Preparation

The preparation of the DNA carrier with an aptamer was performed in three steps. First, we prepared a DNA gap constructed from a 9 kb plasmid. By adding restriction sites, we formed a 40-base-long single-strand region (5′-GGGCGTACCTTTTTCTCGATATCGCATCTCCGCAAGCTGA-3′) in the middle of the sequence and biotin at one end (construction from the plasmid is described in SI). The gap was located at a distance of 3670 bp from the end with biotin for the DNA with a single gap, while the DNA with two gaps has another gap 1030 bp further along the molecule. Note that when designing the DNA carrier, the aptamer binding site should be positioned at least 1500 bp away from the biotin bound to the surface. The capture distance between the substrate’s surface and the nanopore as well as DNA capture rates varies across experiments. It depends on factors such as nanopore size, applied bias, and the concentration and type of ionic species in the buffer.^60^ Second, we folded the aptamer part of the extended TBA 1 sequences (5′-TCAGCTTGCGGAGATGCGAT GGTTGGTGTGGTTGG ATCGAGAAAAAGGTACGCCC-3′) into their active tertiary structure. The activity of the aptamer in the presence of dsDNA was checked with a gel. The extended TBA 1 sequences were folded and annealed to the oligo-DNA with a Cy5 dye (the sequence matches that of the gap). After that, a mixture of thrombin protein was added in different concentrations and run through 16% Tris-Glycine gel to check the binding rate (see Figure S1B,C). This confirmed that thrombin does bind to antithrombin even in the presence of dsDNA. Third, we hybridized the extended TBA 1 sequence to the DNA gap structure. Initially, the aptamer was folded by annealing it from 95 °C. Afterward, we mixed the folded aptamers with the DNA gap structure and performed a second annealing step at 65 °C (see Figure S1A). The same protocol was applied to the TBA 2 aptamer, which has the sequence (5′-TCAGCTTGCGGAGATGCGAT AGTCCGTGGTAGGGCAGGTTGGGGTGACT ATCGAGAAAAAGGTACGCCC-3′). After the analyte was prepared, we tethered it to the surface and allowed it to incubate for several days. The distribution of the analyte on the surface was verified using fluorescence imaging (Figure 1 insertion).

#### Preparation of DNA Carrier with 1 Gap

A 9 kb plasmid was genetically modified in order to introduce sequences for primers and restriction sites (NtBbvCI and EcorV) to generate a template in which the NtBbvCI sequence was 40 bp away. The plasmid was linearized by PCR reaction with the following primers in order to introduce a Biotin at 5′: Fwd TCCCTAGGGCGGCCGCGAATTAA and Rev ATTCTACGTAAGCTTCAGCCTCTCTTTTC 5′ Modification BIOTIN. After purifying the PCR product, the DNA was digested with Nt BbvCI nicking enzymes for 3h at 37C. This RE cut only one of the two strands leaving a gap. The nicked plasmid was incubated with a 100X molar excess of a single-stranded DNA competitor and thermally treated with the following thermal protocol: 90 °C (5 min), 80 °C (1 min) and −1 °C touchdown (1 min, 30x), 50 °C (30 min), and −1 °C touchdown (1 min, 30x). Additional digestion with EcoRV for 2h at 37 C allowed selective cutting of double-stranded DNA, leaving full size only the nicked constructs. The gapped DNA constructs were isolated and extracted by Agarose gel (1%) electrophoresis.

#### Preparation of DNA Carrier with 2 Gaps

In order to create a DNA molecule with two 40 nt gaps approximately 1000 bp apart and with the first gap located at 3.6 kb from the biotin tail, a 9 kb plasmid was genetically modified to introduce restriction sites for the Nt.BbvCI and EcoRV enzymes. The plasmid was linearized and the biotin was introduced via PCR. After purification of the PCR product, the DNA was digested with Nt BbvCI nicking enzyme, which hydrolyzes only one of the two strands of the DNA molecule. The nicked plasmid was incubated with a 100X molar excess of a single-stranded DNA competitor (two 40 nt DNA oligonucleotides, one for each gap), and finally, the product was digested with EcoRV enzyme, which digests only the DNA fragments without gaps (double-stranded). After migration on a 1% agarose gel, bands corresponding to the gapped plasmid were extracted with a gel extraction kit.

#### Immobilisation of the Surface with DNA

We used chambers with a glass surface that can contain between 5 and 400 uL of liquid. Before the surface treatment was started, the chamber was plasma-cleaned to achieve full glass-surface coverage. Initially, we fill half of the full chamber volume with phosphate-buffered saline (PBS) and the other half with 1 mg mL^–1^ of biotin-conjugated bovine serum albumin (Sigma-Aldrich). After incubating for at least 1 h, the chamber is washed 10× with PBS, by exchanging half of the solution each time. Next, half of the volume was removed and replaced with 0.1 mg mL^–1^ streptavidin (Sigma-Aldrich) for a 1 h incubation, followed by another 10× wash with PBS. Finally, one-half of the volume is taken out and placed in DNA carriers of concentration 100–200 pM. The samples are stored at 4 °C until use. Before experiments, the sample was washed 10x with a buffer.

Note that care must be taken to ensure that the sample is not fully dry. In order to reuse the bound DNA carriers, we recommend a stepwise dilution whereby only half the buffer volume is replaced at a single step and for this dilution to be repeated 20–30 times. For long-term storage, we used a parafilm-sealed container and kept the sample at 4 °C.

#### Single-Molecule Fluorescence Imaging

To check and optimize the surface density of attached DNA carriers, we used YO-PRO-1 dye (Thermo Fisher Scientific). After incubating the sample for 30 min, we imaged the surface in TIRF mode with a 488 nm laser. The resulting image is inserted in Figure 1A.

#### Solutions and Reagents

Thrombin was obtained from Sigma-Aldrich Thrombin from human plasma 20 U and diluted in 1 M KCl or 150 mM KCL buffer at pH 7.5. Human serum was purchased from Sigma-Aldrich, Human serum type AB - male. Thrombin at a concentration of 600 nM (in 1 M KCl) was introduced to the sample and incubated for 30 min. This concentration is three-fold higher than the dissociation constant (*K*_d_) value to ensure complete binding. In the measurement with human serum, a thrombin concentration of 200 nM was introduced.

## Supporting Information Available

Gel images showing the hybridization of the aptamer to the DNA carrier, SEM images of the capillary used for measurements, ionic currents traces of controlled translocation of both aptamer and aptamer-bound thrombin molecules in 1 M KCl, results from free translocation experiments on DNA carriers with one aptamer, and table of RMS values for ionic current traces corresponding to the thrombin molecule at different scanning speeds (PDF)

## References

[ref1] Hornblower B.; et al. Single-molecule analysis of DNA-protein complexes using nanopores. Nat. Methods 2007, 4, 315–317. 10.1038/nmeth1021. 17339846

[ref2] Plesa C.; et al. Fast Translocation of Proteins through Solid State Nanopores. Nano Lett. 2013, 13, 658–663. 10.1021/nl3042678. 23343345 PMC4151282

[ref3] Bell N. A. W.; Keyser U. F. Specific Protein Detection Using Designed DNA Carriers and Nanopores. J. Am. Chem. Soc. 2015, 137, 2035–2041. 10.1021/ja512521w. 25621373 PMC4353036

[ref4] Sze J. Y. Y.; Ivanov A. P.; Cass A. E. G.; Edel J. B. Single molecule multiplexed nanopore protein screening in human serum using aptamer modified DNA carriers. Nat. Commun. 2017, 8, 1552 10.1038/s41467-017-01584-3. 29146902 PMC5691071

[ref5] Xue L.; et al. Solid-state nanopore sensors. Nat. Rev. Mater. 2020, 5, 931–951. 10.1038/s41578-020-0229-6.

[ref6] Houghtaling J.; List J.; Mayer M. Nanopore-Based, Rapid Characterization of Individual Amyloid Particles in Solution: Concepts, Challenges, and Prospects. Small 2018, 14, 1802412 10.1002/smll.201802412. 30225962

[ref7] Li W.; et al. Single Protein Molecule Detection by Glass Nanopores. ACS Nano 2013, 7, 4129–4134. 10.1021/nn4004567. 23607870

[ref8] Steinbock L. J.; Otto O.; Chimerel C.; Gornall J.; Keyser U. F. Detecting DNA Folding with Nanocapillaries. Nano Lett. 2010, 10, 2493–2497. 10.1021/nl100997s. 20515038

[ref9] Zrehen A.; Huttner D.; Meller A. On-Chip Stretching, Sorting, and Electro-Optical Nanopore Sensing of Ultralong Human Genomic DNA. ACS Nano 2019, 13, 14388–14398. 10.1021/acsnano.9b07873. 31756076 PMC6933818

[ref10] Kowalczyk S. W.; Tuijtel M. W.; Donkers S. P.; Dekker C. Unraveling Single-Stranded DNA in a Solid-State Nanopore. Nano Lett. 2010, 10, 1414–1420. 10.1021/nl100271c. 20235508

[ref11] Manrao E. A.; et al. Reading DNA at single-nucleotide resolution with a mutant MspA nanopore and phi29 DNA polymerase. Nat. Biotechnol. 2012, 30, 349–353. 10.1038/nbt.2171. 22446694 PMC3757088

[ref12] Platnich C. M.; Earle M. K.; Keyser U. F. Chemical Annealing Restructures RNA for Nanopore Detection. J. Am. Chem. Soc. 2024, 146, 12919–12924. 10.1021/jacs.4c03753. 38691627 PMC11099964

[ref13] Bošković F.; et al. Nanopore Translocation Reveals Electrophoretic Force on Noncanonical RNA:DNA Double Helix. ACS Nano 2024, 18, 15013–15024. 10.1021/acsnano.4c01466. 38822455 PMC11171748

[ref14] Butler T. Z.; Gundlach J. H.; Troll M. A. Determination of RNA Orientation during Translocation through a Biological Nanopore. Biophys. J. 2006, 90, 190–199. 10.1529/biophysj.105.068957. 16214857 PMC1367018

[ref15] Rozevsky Y.; et al. Quantification of mRNA Expression Using Single-Molecule Nanopore Sensing. ACS Nano 2020, 14, 13964–13974. 10.1021/acsnano.0c06375. 32930583 PMC7510349

[ref16] Cai S.; et al. Selective Single-Molecule Nanopore Detection of mpox A29 Protein Directly in Biofluids. Nano Lett. 2023, 23, 11438–11446. 10.1021/acs.nanolett.3c02709. 38051760 PMC10755749

[ref17] Freedman K. J.; et al. Chemical, Thermal, and Electric Field Induced Unfolding of Single Protein Molecules Studied Using Nanopores. Anal. Chem. 2011, 83, 5137–5144. 10.1021/ac2001725. 21598904

[ref18] Yusko E. C.; et al. Controlling protein translocation through nanopores with bio-inspired fluid walls. Nat. Nanotechnol. 2011, 6, 253–260. 10.1038/nnano.2011.12. 21336266 PMC3071889

[ref19] Motone K.; et al. Multi-pass, single-molecule nanopore reading of long protein strands. Nature 2024, 633, 662–669. 10.1038/s41586-024-07935-7. 39261738 PMC11410661

[ref20] Cao C.; et al. Discrimination of oligonucleotides of different lengths with a wild-type aerolysin nanopore. Nat. Nanotechnol. 2016, 11, 713–718. 10.1038/nnano.2016.66. 27111839

[ref21] Cao C.; et al. Deep Learning-Assisted Single-Molecule Detection of Protein Post-translational Modifications with a Biological Nanopore. ACS Nano 2024, 18, 1504–1515. 10.1021/acsnano.3c08623. 38112538 PMC10795472

[ref22] Larkin J.; Henley R. Y.; Muthukumar M.; Rosenstein J. K.; Wanunu M. High-Bandwidth Protein Analysis Using Solid-State Nanopores. Biophys. J. 2014, 106, 696–704. 10.1016/j.bpj.2013.12.025. 24507610 PMC3944622

[ref23] Luo Y.; Wu L.; Tu J.; Lu Z. Application of Solid-State Nanopore in Protein Detection. Int. J. Mol. Sci. 2020, 21, 2808 10.3390/ijms21082808. 32316558 PMC7215903

[ref24] Robertson J. W. F.; Reiner J. E. The Utility of Nanopore Technology for Protein and Peptide Sensing. Proteomics 2018, 18, e1800026 10.1002/pmic.201800026. 29952121 PMC10935609

[ref25] Sandler S. E.; et al. Multiplexed Digital Characterization of Misfolded Protein Oligomers via Solid-State Nanopores. J. Am. Chem. Soc. 2023, 145, 25776–25788. 10.1021/jacs.3c09335. 37972287 PMC10690769

[ref26] Kowalczyk S. W.; Hall A. R.; Dekker C. Detection of Local Protein Structures along DNA Using Solid-State Nanopores. Nano Lett. 2010, 10, 324–328. 10.1021/nl903631m. 19902919

[ref27] Yang W.; et al. Detection of CRISPR-dCas9 on DNA with Solid-State Nanopores. Nano Lett. 2018, 18, 6469–6474. 10.1021/acs.nanolett.8b02968. 30187755 PMC6187524

[ref28] Bulushev R. D.; Marion S.; Radenovic A. Relevance of the Drag Force during Controlled Translocation of a DNA–Protein Complex through a Glass Nanocapillary. Nano Lett. 2015, 15, 7118–7125. 10.1021/acs.nanolett.5b03264. 26393370

[ref29] Ying Y.-L.; Cao C.; Long Y.-T. Single molecule analysis by biological nanopore sensors. Analyst 2014, 139, 3826–3835. 10.1039/C4AN00706A. 24991734

[ref30] Ying Y.-L.; et al. Nanopore-based technologies beyond DNA sequencing. Nat. Nanotechnol. 2022, 17, 1136–1146. 10.1038/s41565-022-01193-2. 36163504

[ref31] Fragasso A.; Schmid S.; Dekker C. Comparing Current Noise in Biological and Solid-State Nanopores. ACS Nano 2020, 14, 1338–1349. 10.1021/acsnano.9b09353. 32049492 PMC7045697

[ref32] Wanunu M.; Morrison W.; Rabin Y.; Grosberg A. Y.; Meller A. Electrostatic focusing of unlabelled DNA into nanoscale pores using a salt gradient. Nat. Nanotechnol. 2010, 5, 160–165. 10.1038/nnano.2009.379. 20023645 PMC2849735

[ref33] Huang G.; Willems K.; Soskine M.; Wloka C.; Maglia G. Electro-osmotic capture and ionic discrimination of peptide and protein biomarkers with FraC nanopores. Nat. Commun. 2017, 8, 935 10.1038/s41467-017-01006-4. 29038539 PMC5715100

[ref34] Zhang S.; et al. Bottom-up fabrication of a proteasome–nanopore that unravels and processes single proteins. Nat. Chem. 2021, 13, 1192–1199. 10.1038/s41557-021-00824-w. 34795436 PMC7612055

[ref35] Squires A. H.; Hersey J. S.; Grinstaff M. W.; Meller A. A Nanopore–Nanofiber Mesh Biosensor To Control DNA Translocation. J. Am. Chem. Soc. 2013, 135, 16304–16307. 10.1021/ja408685x. 24143914 PMC4039743

[ref36] Ohayon S.; et al. Full-Length Single Protein Molecules Tracking and Counting in Thin Silicon Channels. Adv. Mater. 2024, 36, 2314319 10.1002/adma.202314319. 38461367

[ref37] Schmid S.; Stömmer P.; Dietz H.; Dekker C. Nanopore electro-osmotic trap for the label-free study of single proteins and their conformations. Nat. Nanotechnol. 2021, 16, 1244–1250. 10.1038/s41565-021-00958-5. 34462599

[ref38] Eggenberger O. M.; Ying C.; Mayer M. Surface coatings for solid-state nanopores. Nanoscale 2019, 11, 19636–19657. 10.1039/C9NR05367K. 31603455

[ref39] Ren R.; et al. Multiplexed detection of viral antigen and RNA using nanopore sensing and encoded molecular probes. Nat. Commun. 2023, 14, 7362 10.1038/s41467-023-43004-9. 37963924 PMC10646045

[ref40] Bošković F.; et al. Simultaneous identification of viruses and viral variants with programmable DNA nanobait. Nat. Nanotechnol. 2023, 18, 290–298. 10.1038/s41565-022-01287-x. 36646828 PMC10020084

[ref41] Bell N. A. W.; et al. DNA Origami Nanopores. Nano Lett. 2012, 12, 512–517. 10.1021/nl204098n. 22196850

[ref42] Leitao S. M.; et al. Spatially multiplexed single-molecule translocations through a nanopore at controlled speeds. Nat. Nanotechnol. 2023, 18, 1078–1084. 10.1038/s41565-023-01412-4. 37337057

[ref43] Plesa C.; Van Loo N.; Ketterer P.; Dietz H.; Dekker C. Velocity of DNA during Translocation through a Solid-State Nanopore. Nano Lett. 2015, 15, 732–737. 10.1021/nl504375c. 25496458

[ref44] Chen K.; et al. Dynamics of driven polymer transport through a nanopore. Nat. Phys. 2021, 17, 1043–1049. 10.1038/s41567-021-01268-2.

[ref45] Plesa C.; et al. Direct observation of DNA knots using a solid-state nanopore. Nat. Nanotechnol. 2016, 11, 1093–1097. 10.1038/nnano.2016.153. 27525473

[ref46] Pud S.; et al. Mechanical Trapping of DNA in a Double-Nanopore System. Nano Lett. 2016, 16, 8021–8028. 10.1021/acs.nanolett.6b04642. 27960493 PMC5523128

[ref47] ChouY.-C.; et al. Coupled nanopores for single-molecule detection. Nat. Nanotechnol. 2024, 19, 1686–1692. 10.1038/s41565-024-01746-7.39143316

[ref48] Macaya R. F.; Schultze P.; Smith F. W.; Roe J. A.; Feigon J. Thrombin-binding DNA aptamer forms a unimolecular quadruplex structure in solution. Proc. Natl. Acad. Sci. U. S. A. 1993, 90, 3745–3749. 10.1073/pnas.90.8.3745. 8475124 PMC46378

[ref49] Paborsky L. R.; McCurdy S. N.; Griffin L. C.; Toole J. J.; Leung L. L. The single-stranded DNA aptamer-binding site of human thrombin. J. Biol. Chem. 1993, 268, 20808–20811. 10.1016/S0021-9258(19)36856-5. 8407909

[ref50] Tasset D. M.; Kubik M. F.; Steiner W. Oligonucleotide inhibitors of human thrombin that bind distinct epitopes1. J. Mol. Biol. 1997, 272, 688–698. 10.1006/jmbi.1997.1275. 9368651

[ref51] Deng B.; et al. Aptamer binding assays for proteins: the thrombin example--a review. Anal. Chim. Acta 2014, 837, 1–15. 10.1016/j.aca.2014.04.055. 25000852

[ref52] Leitao S. M.; et al. Time-Resolved Scanning Ion Conductance Microscopy for Three-Dimensional Tracking of Nanoscale Cell Surface Dynamics. ACS Nano 2021, 15, 17613–17622. 10.1021/acsnano.1c05202. 34751034 PMC8613909

[ref53] Plesa C.; Dekker C. Data analysis methods for solid-state nanopores. Nanotechnology 2015, 26, 084003 10.1088/0957-4484/26/8/084003. 25648179

[ref54] Wang M. D.; Yin H.; Landick R.; Gelles J.; Block S. M. Stretching DNA with optical tweezers. Biophys. J. 1997, 72, 1335–1346. 10.1016/S0006-3495(97)78780-0. 9138579 PMC1184516

[ref55] Bennink M. L.; et al. Single-molecule manipulation of double-stranded DNA using optical tweezers: Interaction studies of DNA with RecA and YOYO-1. Cytometry 1999, 36, 200–208. 10.1002/(SICI)1097-0320(19990701)36:3<200::AID-CYTO9>3.0.CO;2-T. 10404969

[ref56] Bennett H.-A.; Li Y.; Yan H. Thermal treatment affects aptamers’ structural profiles. Bioorg. Med. Chem. Lett. 2023, 82, 129150 10.1016/j.bmcl.2023.129150. 36693483

[ref57] Smirnov I.; Kolganova N.; Troisi R.; Sica F.; Timofeev E. Expanding the recognition interface of the thrombin-binding aptamer HD1 through modification of residues T3 and T12. Molecular Therapy - Nucleic Acids 2021, 23, 863–871. 10.1016/j.omtn.2021.01.004. 33614235 PMC7868722

[ref58] Tan S. Y.; Acquah C.; Tan S. Y.; Ongkudon C. M.; Danquah M. K. Characterisation of charge distribution and stability of aptamer-thrombin binding interaction. Process Biochemistry 2017, 60, 42–51. 10.1016/j.procbio.2017.06.003.

[ref59] Pagano B.; Martino L.; Randazzo A.; Giancola C. Stability and Binding Properties of a Modified Thrombin Binding Aptamer. Biophys. J. 2008, 94, 562–569. 10.1529/biophysj.107.117382. 17890401 PMC2157226

[ref60] Zhu C.; Huang K.; Siepser N. P.; Baker L. A. Scanning Ion Conductance Microscopy. Chem. Rev. 2021, 121, 11726–11768. 10.1021/acs.chemrev.0c00962. 33295182 PMC8187480

[ref61] Russo Krauss I.; Pica A.; Merlino A.; Mazzarella L.; Sica F. Duplex–quadruplex motifs in a peculiar structural organization cooperatively contribute to thrombin binding of a DNA aptamer. Acta Cryst. D 2013, 69, 2403–2411. 10.1107/S0907444913022269. 24311581

[ref62] Tomasello G.; Armenia I.; Molla G. The Protein Imager: a full-featured online molecular viewer interface with server-side HQ-rendering capabilities. Bioinformatics 2020, 36, 2909–2911. 10.1093/bioinformatics/btaa009. 31930403

