# Supplementary material for: Controlled Sensing of User-Defined Aptamer-Based Targets Using Scanning Ionic Conductance Spectroscopy
Source: ACS Nano. 2025 Mar 31;19(13):13139–48. doi: 10.1021/acsnano.4c18509 (PMC11984302; doi:10.1021/acsnano.4c18509)
Supplement: Supplementary file 1 — nn4c18509_si_001.pdf [file nn4c18509_si_001.pdf]

Supporting information

Controlled sensing of user defined aptamer-based targets using Scanning Ionic Conductance Spectroscopy

Helena Miljkovic[1,2], Lely Feletti[1], Gordanna Pistoletti Blanchet[3], Marcos Penedo[4], Zahra Ayar[4], Barney Drake[4],Alexandre Kuhn[3], Wayne Yang[1,*], Georg E. Fantner[4,*], Aleksandra Radenovic[1,2,*]

[1] *Laboratory of Nanoscale Biology (LBEN), Institute of Bioengineering, School of Engineering, Swiss Federal Institute of Technology Lausanne (EPFL), Lausanne 1015, Switzerland.*

[2] *NCCR Bio-inspired Materials, École Polytechnique Fédérale de Lausanne, Lausanne 1015, Switzerland.*

[3] *Laboratory of Molecular Biology, Institute of Life Sciences, School of Engineering, HES-SO Valais-Wallis, Sion 1950, Switzerland.*

[4] *Laboratory for Bio and Nano Instrumentation (LBNI), Institute of Bioengineering, School of Engineering, Swiss Federal Institute of Technology Lausanne (EPFL), Lausanne 1015, Switzerland.*

[*] Corresponding Authors

e-mail : wayne.yang@epfl.ch , georg.fantner@epfl.ch , and aleksandra.radenovic@epfl.ch

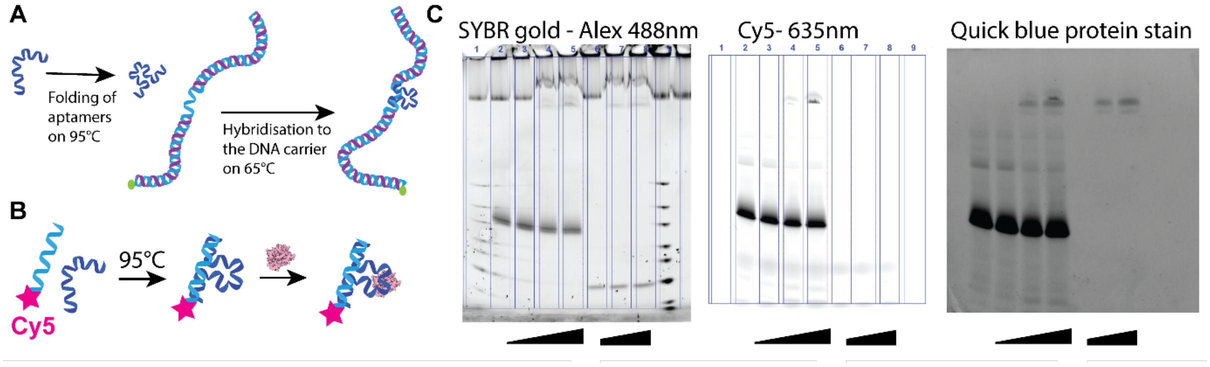

SI Figure 1. <u>Hybridisation of aptamer to the DNA carrier</u>. **A** The aptamer was folded by annealing from 95°C to 4°C. Hybridization to the DNA carrier was done through annealing process from 65°C to 4°C. **B** Folding of extended aptamer sequence and hybridisation to the sequence of the gap of DNA carrier with attached Cy5 dye. After annealing procedure from 95C to 4C we introduced thrombin to the sample. **C** Image of the 16% Tris-Glycine gel. **Lanes 1 and 10** - 100b ladders. **Lanes 2,3,4 and 5** - folded aptamer with attached ssDNA with Cy5 dye. The concentration gradient increases sequentially 0.1x, 1x, 10x, and 100x. Concentration of aptamer oligo is 1nM. **Lanes 6, 7, 8** - folded aptamer sequences. The concentration gradient increases sequentially 1x, 10x, and 100x. Concentration of aptamer oligo is 1nM.

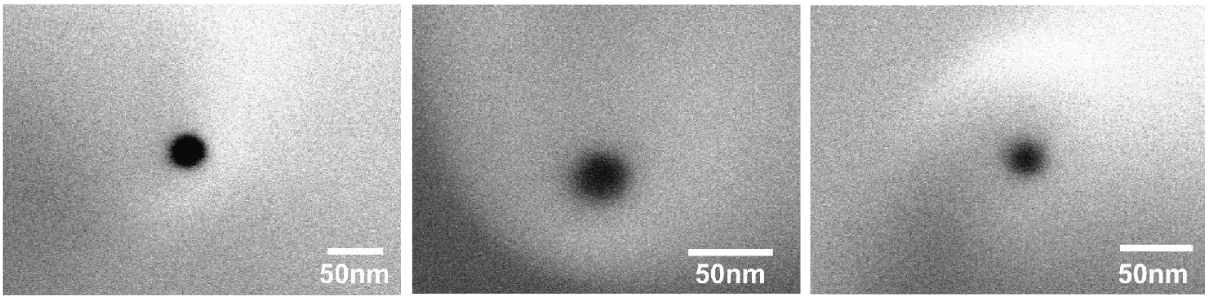

SI Figure 2. **<u>SEM image of the capillary used for the measurements.</u>**

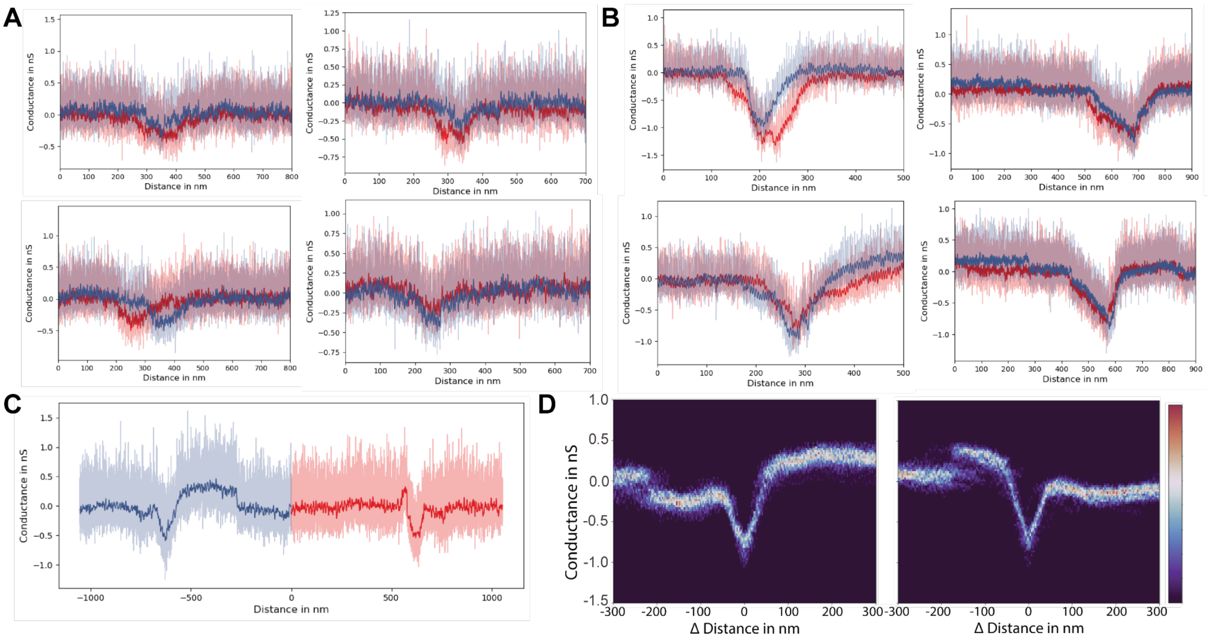

SI Figure 3. <u>Controlled translocation of aptamer and aptamer bound thrombin molecules in 1M KCl.</u> **A** Examples of aptamer traces. **B** Examples of thrombin traces. **C** Trace of aptamer attached only on one side. **D** Density function plot of repeated measurements of the same molecule (gap 1, TBA1) with the same capillary (N=17). Left: Density function plot of forward scans. Right: Density function plot of backward scans.

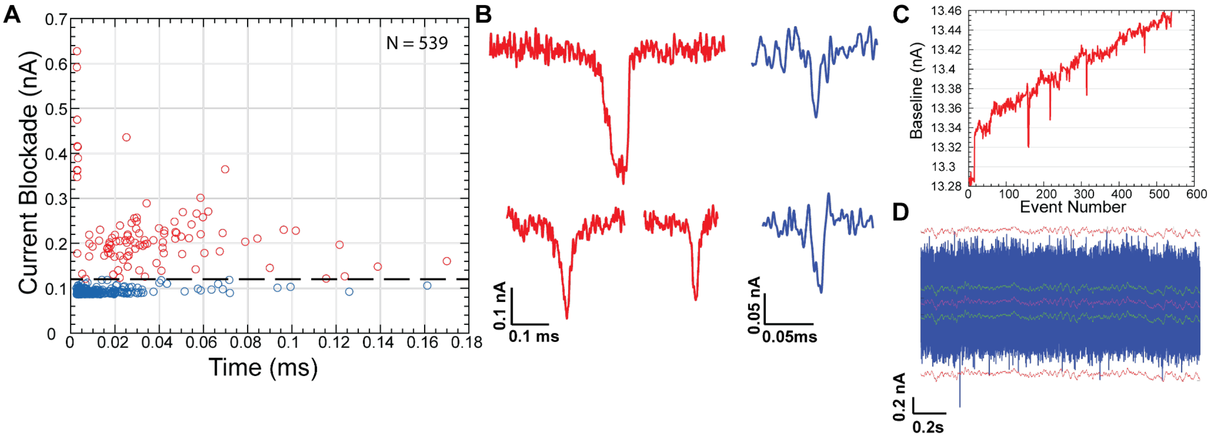

SI Figure 4. **<u>Free translocation experiments on DNA carriers with one aptamer.</u>** **A** Scatter plot of the event (N=539) gathered in 1M KCl and 300mV. Dotted line separates protein bound events (above, red) from DNA events without proteins bound (below, blue). **B** Examples of traces. Red: protein bound events. Blue: DNA carrier without any protein. **C** Baseline of detected events. **D** Example of baseline current during the measurements. Data is analysed with tantalizer.

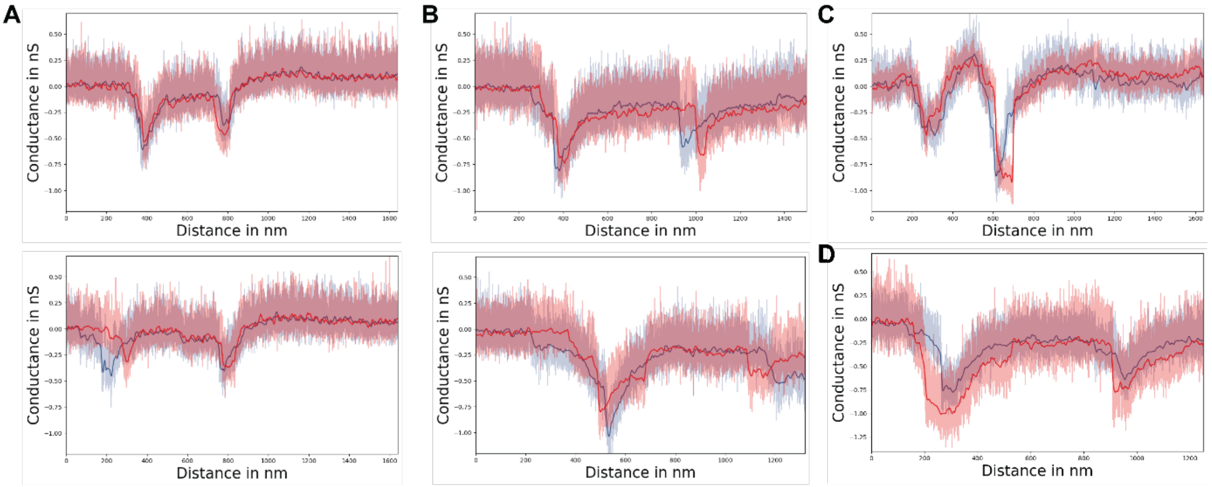

SI Figure 5. <u>Controlled translocation of 2 aptamer DNA construct in 1M KCl.</u> A Examples of traces with only aptamers. **B** Examples of traces with thrombin on TBA2. **C** Example of a trace with thrombin on TBA1. **D** Examples of a trace with thrombin on TBA1 and TBA2.

Set scanner speed	RMS
100 nm/s	0.23
300 nm/s	0.27
500 nm/s	0.31
1000 nm/s	0.33

Table 1. RMS of ionic current traces for the thrombin molecule at different scanning speeds.
